# Developing Antimicrobial Synergy With AMPs

**DOI:** 10.3389/fmedt.2021.640981

**Published:** 2021-03-12

**Authors:** Leora Duong, Steven P. Gross, Albert Siryaporn

**Affiliations:** ^1^Department of Molecular Biology & Biochemistry, University of California, Irvine, Irvine, CA, United States; ^2^Department of Developmental and Cell Biology, University of California, Irvine, Irvine, CA, United States; ^3^Department of Physics & Astronomy, University of California, Irvine, Irvine, CA, United States

**Keywords:** antimicrobial peptides, histones, antimicrobial synergism, antibiotic resistance, intracellular targeting

## Abstract

Antimicrobial peptides (AMPs) have been extensively studied due to their vast natural abundance and ability to kill microbes. In an era critically lacking in new antibiotics, manipulating AMPs for therapeutic application is a promising option. However, bacterial pathogens resistant to AMPs remain problematic. To improve AMPs antimicrobial efficacy, their use in conjunction with other antimicrobials has been proposed. How might this work? AMPs kill bacteria by forming pores in bacterial membranes or by inhibiting bacterial macromolecular functions. What remains unknown is the duration for which AMPs keep bacterial pores open, and the extent to which bacteria can recover by repairing these pores. In this mini-review, we discuss various antimicrobial synergies with AMPs. Such synergies might arise if the antimicrobial agents helped to keep bacterial pores open for longer periods of time, prevented pore repair, perturbed bacterial intracellular functions at greater levels, or performed other independent bacterial killing mechanisms. We first discuss combinations of AMPs, and then focus on histones, which have antimicrobial activity and co-localize with AMPs on lipid droplets and in neutrophil extracellular traps (NETs). Recent work has demonstrated that histones can enhance AMP-induced membrane permeation. It is possible that histones, histone fragments, and histone-like peptides could amplify the antimicrobial effects of AMPs, giving rise to antimicrobial synergy. If so, clarifying these mechanisms will thus improve our overall understanding of the antimicrobial processes and potentially contribute to improved drug design.

## Introduction

Bacterial infections are an increasing threat to global health, due to both an increase in bacterial resistance to current therapeutics and also a decline in new antibiotic development. This results in rising numbers of untreatable health complications and deaths worldwide (1). There is thus an urgent need to identify new antibacterial strategies to effectively treat drug-resistant pathogens. The demand for such new strategies has encouraged scientists to investigate biologically-abundant antimicrobial tools that can be manipulated to kill bacteria. Repurposing and modifying known natural antimicrobial proteins may contribute to successful development of new therapeutic strategies.

Antimicrobial peptides (AMPs) have broad spectrum antimicrobial activity and are found ubiquitously in nature. They have been extensively studied as a promising option to combat multidrug-resistant bacteria. However, the rapid ability of bacteria to evolve requires new approaches to limit potential bacterial resistance to AMPs (2–4). Here, we discuss the use of AMPs in conjunction with other antimicrobials to form antimicrobial synergy, in which the combined antimicrobial effect is greater than the sum of either treatment alone. Antimicrobial synergy could potentially reduce the rise of bacterial resistance. A number of synergistic approaches using AMPs have been sought, with 300 reports made during the last 5 years as determined by PubMed. We examine and propose potential mechanisms that give rise to antimicrobial synergy with AMPs.

## Physiological Roles

AMPs are ubiquitously observed in nature and are known for their physiological antimicrobial roles. They are produced by both prokaryotic and eukaryotic organisms, ranging from bacteria (5, 6), insects (7–9), amphibians (10–12), and humans (13–16). AMPs protect organisms from microbial harm and thus play vital roles in innate immunity (17) by directly or indirectly killing microbes. AMPs directly kill microbes by acting at the bacterial membrane (18, 19) or eliciting bacterial cell death via inhibition of macromolecular functions (20). AMPs indirectly kill microbes by directing cytokines to sites of infection for increased immunological responses in hosts (21). Neutrophils, the first line of innate immune defense, have dense granules that are packed with AMPs that are used to defend against microbial infections (22). When stimulated, neutrophils can also release their intracellular contents to form neutrophil extracellular traps (NETs). These web-like structures, consisting of DNA, AMPs, and other antimicrobial agents, can entrap and kill bacteria (23, 24). Similar to neutrophil elastases, AMPs have vital roles in NETs in controlling microbial threats (25). A recent report indicates that AMPs also localize to cellular lipid droplets with histones (26) and contribute to lipid-droplet based cellular immunity.

## Structure and Function

AMPs are typically small peptides, ranging from about 5 to 50 amino acids, but can be as large as over 100 amino acids (27). Most AMPs are positively charged (+2 to +9) due to their high proportions of arginine and lysine residues (28), though negatively charged AMPs do also exist (21, 29, 30). Structures of AMPs include α-helix, β-sheet, extended, and loop (31), with α-helix and β-sheet structures being the most common. More complex structures also exist, including cyclic and lasso peptides (32). AMPs are known for their amphipathic nature, typically consisting 50% of hydrophobic residues including alanine, glycine, and leucine (28, 33). The biophysical properties of AMPs contribute to their potent antimicrobial activity. Cationic (positively charged) AMPs can bind to anionic (negatively charged) lipopolysaccharide (LPS) and lipoteichoic acid (LTA), which are major components of bacterial membranes (34). The amphipathic nature of AMPs also enables them to interact with and insert into bacterial cell membranes.

Many reports attribute the antimicrobial activity of AMPs to the formation of pores within bacterial membranes, which can elicit cell damage and death. Several different classes of AMP-induced membrane pores have been proposed, including barrel-stave, toroidal, and carpet (20). In a barrel-stave model, peptide monomers form a transmembrane channel that is parallel to bacterial membrane phospholipids. A toroidal model proposes that AMPs insert into bacterial cell membranes and force membrane lipid structures to change in conformation, as opposed to pore insertion through an intact membrane like that of the barrel-stave model. The carpet model suggests that AMPs do not form transmembrane pores but instead localize to the bacterial membrane surface, where they disrupt membrane organization and integrity (35). These membrane disruptions can cause loss of bacterial membrane proton gradient, cell leakage, and eventually cell death (19). Alternative models to pore formation in membranes have also been proposed, with pore formation and cell leakage being attributed to the high concentrations of AMPs that are typically used in membrane pore formation studies (35). In particular, the entry of AMPs into bacterial cells may induce intracellular damage, including disruption of bacterial nucleic acid synthesis, protein synthesis, cell wall synthesis, and cell division (20).

## Bacterial Resistance to AMPs

LPS in Gram-negative bacterial membranes and LTA in Gram-positive cell walls contribute to overall negative charges of bacterial cell exteriors. Negatively charged membranes, which are conserved among bacteria, provide cytoplasmic rigidity and proper cationic gradients that are necessary for bacterial survival (36). However, cationic AMPs can easily bind to anionic components of bacterial membranes via electrostatic interactions to elicit cell damage. Complete bacterial resistance to AMPs is unlikely because evolving a bacterial membrane that possesses an outer neutral or positive charge simply for the purpose of avoiding AMPs would be too evolutionarily costly (37, 38). Still, many studies have shown that bacteria can have intrinsic resistance or evolve resistance to AMPs (2–4, 39, 40).

A vast array of bacterial resistance and defense mechanisms against AMPs exist, including the utilization of efflux pumps (41–43), modifications to cell membrane charge (38), expression of protective barriers around bacterial membranes (44), inhibition of antimicrobials via peptide cleavage (45, 46), and potential membrane healing and recovery post-damage (47). Both multidrug-resistant Gram-negative and Gram-positive bacteria utilize efflux pump mechanisms to actively pump AMPs back out into the extracellular environment to prevent cell damage (41, 42). In Gram-negative *S*. Typhimurium and *P. aeruginosa*, the lipid A portion of LPS is modified with the addition of 4-amino-4-deoxy-L-arabinose, which reduces the overall negative charge and thus reduces the binding affinity of positively charged AMPs, including azurocidin, polymyxin B (PMB), indolicidin, and LL-37 (48–50). In Gram-positive *S. aureus*, lysine is added to membrane phospholipids, reducing the overall anionic charge and affinity to defensin-like cationic AMPs (51). Colanic acid is a polysaccharide which functions as a protective capsule around many *Enterobacteriaceae* (52) and may prevent AMP-mediated activity. It has been suggested that these capsular polysaccharides play a role in bacterial resistance (40, 53) and virulence (54, 55). For example, capsular polysaccharides increase resistance of *K. pneumoniae, S. pneumoniae*, and *P. aeruginosa* to both PMB and human neutrophil alpha-defensin 1 (53). Additionally, increased slime production by *S. epidermidis* in medical catheters has been reported when bacterial capsular polysaccharides are expressed (54, 55). Bacterial species like *E. coli* and *S*. Typhimurium also release proteases to cleave and inhibit antimicrobials that threaten their survival, particularly protamine and alpha helical cationic AMPs, respectively (45, 46).

Recent work suggests that bacteria can recover from pores formed by LL-37 (47). However, the duration in which AMPs can keep bacterial pores open and the extent to which bacteria can repair these pores is unknown. It is possible that efflux pumps are used to eject AMPs out of the membrane to allow for bacterial lipid bilayers to reform. Additionally, bacterial cell wall biosynthesis may be upregulated for the purpose of membrane repair.

## Antimicrobial Synergies With AMPs

To optimize the use of antibiotics, it is important to mitigate potential bacterial resistance mechanisms. Many AMPs have been tested in clinical trials due their potent antimicrobial activity (56, 57). However, as with any antibiotic, using AMPs is associated with the risk of ever-evolving bacterial resistance that could negate their effects. A potential way to reduce the risk of drug-resistance to AMPs in clinical settings is to use AMPs in conjunction with other antimicrobials, focusing on combinations that lead to effective antimicrobial synergies. Synergistic combinations that have multiple targets in independent pathways could require two independent and simultaneous sets of mutations to address both challenges. Synergy could also be more lethal, decreasing the likelihood that bacteria can escape and develop resistance.

It has been suggested that bacteria are less likely to evolve resistance to antibiotic cocktails than to a single antimicrobial (58, 59). Consistent with this is the fact that multiple AMPs are released during immune responses *in vivo*, making it difficult for bacteria to develop resistance (60). Therefore, using AMP cocktails, especially ones that convey antimicrobial synergy, could be an effective strategy. Synergistic antibacterial combinations with AMPs could enable bacterial pores to stay open for longer durations, prevent pore repair, increase perturbation of bacterial intracellular functions, or convey other independent but complementary bacterial killing mechanisms. These mechanisms may potentially increase antimicrobial efficacy, decrease resistance, and reduce host toxicity if only low concentrations of each antimicrobial component are needed to carry out a large antimicrobial effect (61). The abundance of antimicrobial synergies discovered with AMPs presents exciting possibilities for the potential use of synergistic AMP combinations in clinical settings.

### Synergy With Other AMPs

Numerous reports indicate that AMPs synergize with other AMPs. We discuss antimicrobial synergies of AMPs from organisms like insects, amphibians, and mammals, suggesting that synergistic interactions are common between AMPs within the animal kingdom.

The insect AMPs, diptericins and attacins, show synergistic killing against *P. burhodogranariea* in flies (62). A combination of the synthetic AMP pexiganan and bumblebee AMP melittin show *S. aureus* killing effects comparable to that of Vancomycin, a last line of defense antibiotic (39). Additionally, the antimicrobial activity of a bumblebee AMP, abaecin, is synergistically enhanced by the presence of a pore forming AMP, hymenoptaecin (63). In this example, hymenoptaecin forms membrane pores, potentially causing cell leakage or lytic cell death and enabling the entry of abaecin into bacterial cells. The hymenoptaecin-induced pores may increase the ability for abaecin to access and bind to DnaK, a molecular chaperone, to inhibit bacterial replication (63). Thus, the two AMPs work together to kill bacteria on both a membrane and intracellular level.

AMPs can potentially bind to other AMPs to form more potent antibacterial agents. For example, the amphibian AMPs magainin-2 and peptidyl-glycylleucine-carboxyamide (PGLa) work synergistically to inhibit *E. coli* growth (11). When magainin-2 and PGLa are added together, they form a “supramolecule” to quickly induce bacterial membrane pores and mediate pore stabilization (64). Moreover, it has been reported that PGLa forms an antiparallel dimer that spans the cell membrane where it binds to magainin-2 at the C-terminus (65), forming toroidal pore structures (66). These results are consistent with an additional report in which fused AMPs induce greater killing activities in *S. mutans* than on their own (67). These findings suggest that AMPs can bind other AMPs or other types of antimicrobials to give rise to antimicrobial synergy.

The mammalian AMP protegrin 1 has been reported to exhibit synergistic killing activity with indolicidin, LL-37, and bactenecin against *P. aeruginosa* and *E. coli* (68). Additionally, the combination of indolicidin and bactenecin gives rise to antimicrobial synergy against *E. coli* (68). The combinations of protegrin 1 with LL-37, bactenecin with LL-37, and protegrin 1 with bactenecin are also synergistic against *E. faecalis* (68). Lastly, human platelet-derived synthetic AMP combinations of PD1 through PD4 and Arg-Trp repeats RW1 through RW5 are synergistically antimicrobial in platelets (69).

AMPs can be effective when their mechanisms are complementary, such as in the case of the AMPs coleoptericin and defensin. Coleoptericin contributes to the survival of the mealworm beetle, *Tenebrio molitor*, but does not reduce bacterial load. In contrast, defensin does not improve host survival but reduces bacterial load (70). Their combined use both significantly increases host survival and reduces bacterial load (70). Using multiple AMPs together can thus maintain the independent functions of each AMP, resulting in a more effective treatment strategy.

While many studies demonstrate robust antimicrobial synergies with just two AMPs, synergies with three AMPs reveal even greater effects. For example, while apidaecin functions antagonistically with either pexiganan or LL 19-27 (an analog of LL-37), the triple combination of apidaecin, pexiganan, and LL 19-27 demonstrate strong synergism (58). Synergy was also observed from the combination of human β-defensin, LL-37, and lysozyme, which are produced on the skin, against *S. aureus* and *E. coli* (13). The observation of synergy between these antimicrobials is an example in which natural defense molecules have greater activity in combination rather than individually. Thus, combining natural antimicrobials could yield further discoveries of synergy.

### Synergy With Antibiotics

AMPs can also synergize with antibiotics, and in some cases, overcome antibiotic resistance. The use of AMPs to increase the efficacy of already approved antibiotics appears to be a promising option to combat commonly drug-resistant pathogens. The human AMPs, LL-37 and human β-defensin 3 (HBD3), have antimicrobial synergy with the antibiotics tigecycline, moxifloxacin, piperacillin-tazobactam, and meropenem. Specifically, antibiotic killing against *C. difficile* is improved when both LL-37 and HBD3 are present (71). Lastly, LL 17-29 establishes antimicrobial synergy with the antibiotic chloramphenicol against highly virulent bacterial strains, including methicillin-resistant *S. aureus* and multidrug-resistant *P. aeruginosa* (59).

Combining the AMPs nisin Z, pediocin, or colistin with various antibiotics, including penicillin, ampicillin, or rifampicin, is effective in overcoming antibiotic-resistance in *P. fluorescens* (72). Also, the AMP melamine has synergistic killing activities when paired with ciprofloxacin, a fluoroquinolone antibiotic, against antibiotic-resistant strains of *P. aeruginosa*. This combination may aid in overcoming *P. aeruginosa* resistance to fluoroquinolone antibiotics (73). Synergistic combinations of AMPs with PMB (originally discovered as an AMP), erythromycin, and tetracycline have also been shown. In particular, variants of the AMP indolicidin synergize with the antibiotics PMB, tobramycin, gentamycin, and amikacin (74).

One of the mechanisms by which AMPs improve antibiotic function is by disrupting bacterial membranes to aid in the delivery of antibiotics into the bacterial cytoplasm, where antibiotics can act on intracellular targets. For example, the AMP arenicin-1 synergistically functions with antibiotics including ampicillin, erythromycin, and chloramphenicol to kill *S. aureus, S. epidermis, P. aeruginosa*, and *E. coli* (75). Arenicin-1 assists in the uptake of antibiotics into cells and inhibits bacterial growth via hydroxyl radical formation (75), which suggests complementary mechanisms are at play.

### Synergy With Histones

Histones, more commonly known for their roles in condensing eukaryotic DNA, have antibacterial properties (76, 77). However, the mechanisms by which histones kill bacteria have not previously been understood (78). Since histones are positively charged and have similar structures to that of AMPs, it has been suggested that histones and AMPs have redundant antibacterial roles (79, 80). Histones and AMPs colocalize in innate immunity components, including on cellular lipid droplets and in NETs, suggesting that they could work together to kill microbes (26, 81–83). For fish in particular, fractions of salmon histone H1 have reported antimicrobial synergy with lysozyme and a flounder AMP, pleurocidin (84). Recent work demonstrates that histones H2A and H3 can function with the pore-forming AMPs LL-37 and magainin-2 to produce antibacterial synergy against Gram-positive and Gram-negative bacteria (47). Additionally, H2A and the pore-forming antimicrobial PMB synergistically work together to completely inhibit *E. coli* growth over 24 hours (47). It is important to note that histones must be paired with pore forming AMPs in order for this synergistic model to be effective; histones alone have minimal antimicrobial effects at physiological conditions (47). It is possible that other histones, histone fragments, and histone-like peptides also amplify the antimicrobial effects of AMPs and give rise to antimicrobial synergy.

The mechanism of synergy between AMPs and histones is due the ability of AMPs to form pores in bacterial membranes, enabling histones to enter the bacterial cytoplasm (47, 85). Here, histones inhibit global transcription and reorganize bacterial chromosomes. Furthermore, histones enhance AMP-mediated pores that bacteria otherwise would be able to recover from, leading to reduced cell sizes and increased cytoplasmic leakage (47). The uptake of AMPs and histones into bacterial cells elicits an effective antimicrobial response consistent with a positive feedback loop (47). Importantly, if bacterial intracellular functions, like transcription and translation, are inhibited, this could reduce bacterial cell membrane integrity and repair.

**Figure 1:**
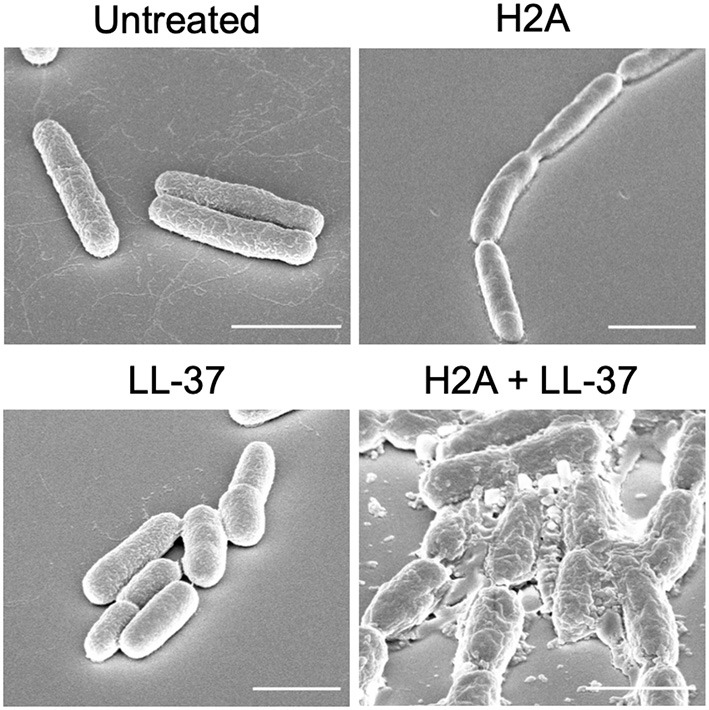
SEM images *E. coli* that are untreated or treated with H2A, LL-37, or both. *E. coli* that are treated with both H2A and LL-37 demonstrate extensive cellular damage. Scale bars indicate 2 μm.

Another potential effect of histones is that they may induce stress on bacterial membranes. This membrane stress could aid AMPs to more effectively form bacterial membrane pores. Altered membrane physiology, revealed through scanning electron microscopy (SEM), suggests that when bacteria are treated with only an individual AMP or histone, the membrane largely remains intact (Figure 1). However, the treatment with both AMPs and histones induces gross cell deformation and leakage of cytoplasmic contents (Figure 1). The reduced membrane integrity from the AMP and histone treatment also inhibits *E. coli* from maintaining their proton gradient, which is necessary for ATP production (47). Thus, membrane damage caused by synergistic combinations with AMPs may lead to lack of recovery from AMP-mediated pores, rapid loss of cytoplasmic content, failure to produce ATP, and ultimately bacterial cell death. In response to histone exposure, the *rcs* gene responsible for colanic acid expression is upregulated in *E. coli* (47). The bacterial upregulation of colanic acid, which functions as a bacterial membrane protective capsule, suggests that there is an active microbial attempt to mitigate potential membrane stress effects due to histones.

### Synergy With Other Antimicrobial Agents

AMPs also synergize with other antimicrobial agents. For example, silver nitrate and silver nanoparticles can synergize with PMB and Gramicidin S, enhancing their intracellular antimicrobial effects in Gram-negative bacteria (86). Additionally, peptoid analogs of AMPs are known to have effective and specific antimicrobial activity (87). AMPs can synergize with peptoids against Gram-negative bacteria (88). The AMP *Galleria mellonella* anionic peptide 2 and antimicrobial enzyme lysozyme are also synergistic against Gram-negative bacteria (89).

## Conclusion

**Figure 2:**
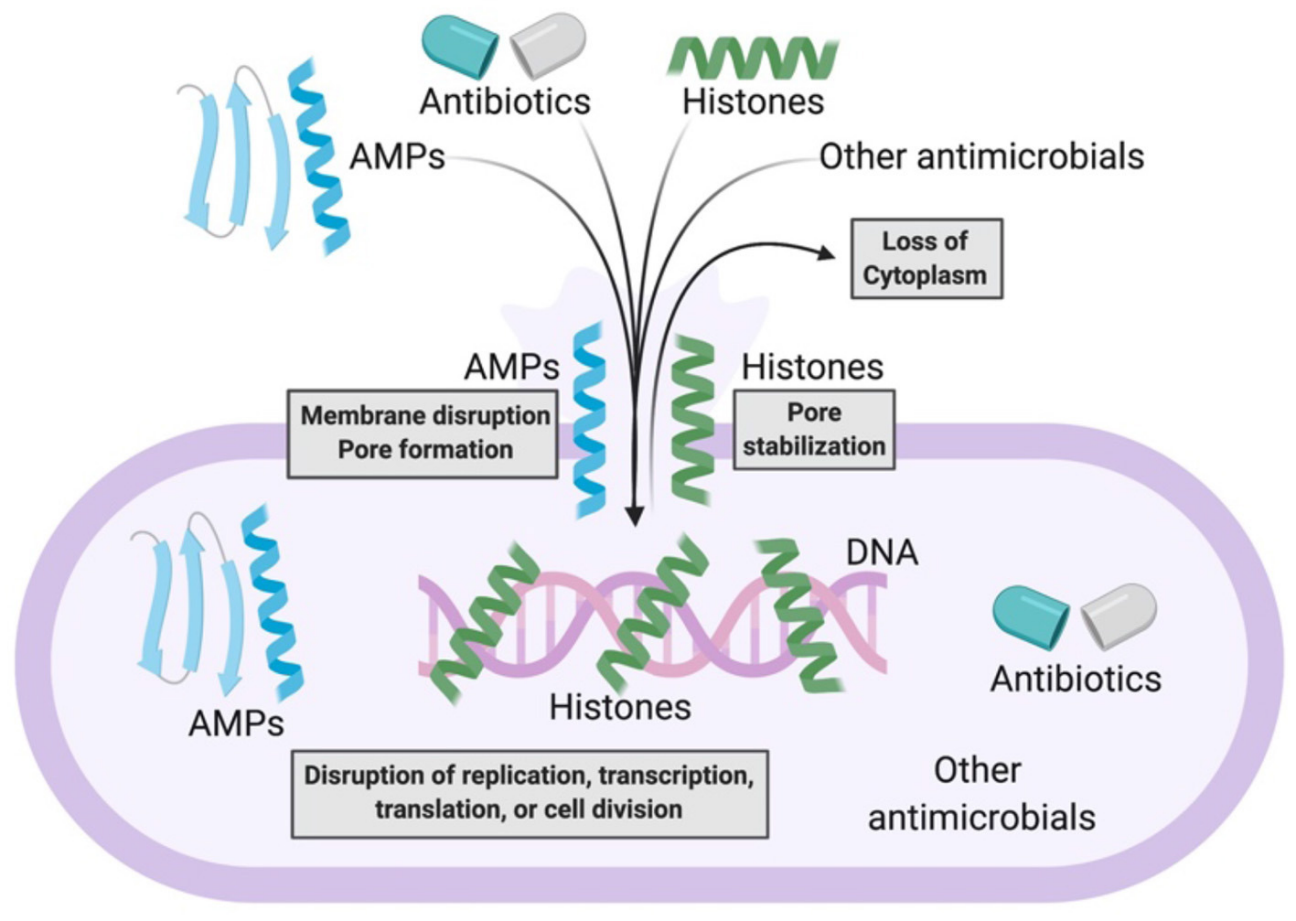
Model of antimicrobial synergy between AMPs and other AMPs, antibiotics, histones, and other antimicrobials. AMPs form bacterial membrane pores or disrupt bacterial membranes. This enables the entry of more AMPs, antibiotics, histones, or other antimicrobials into bacteria. As a result, there is loss of bacterial cytoplasm and disruption of bacterial macromolecular functions. Histones potentially stabilize AMP-induced pores that enable further synergistic antimicrobial activity.

The combination of AMPs with current antimicrobial strategies can produce synergy through a number of distinct mechanisms (Figure 2). The introduction of antibiotics inside bacteria has often been a challenge. However, AMPs can address this challenge by forming membrane pores, thus facilitating entry of antibiotics into the cytoplasm, where the antibiotics can bind to their intracellular targets. The combination of AMPs with antibiotics could thus be an effective antibacterial strategy. This strategy could limit bacterial resistance because defense from the multifaceted attack could be significantly more difficult to achieve.

If the ability for AMPs to synergize with other AMPs or antimicrobials is a conserved characteristic, then relatively low doses of each antimicrobial can be used as antibiotic treatments to exhibit large antimicrobial effects. Lower drug concentrations might also limit harmful side effects. For example, PMB is now an FDA-approved and potent last-resort antibiotic; however, PMB is also highly toxic to the nephrotic and nervous systems (90, 91). Using PMB in a synergistic antimicrobial combination, like with indolicidin or histones, would potentially require lower doses of each antimicrobial agent, potentially reducing host toxicity, while maintaining effective antimicrobial activity. Since the production of peptides can be costly, taking advantage of lower antimicrobial doses needed for synergistic treatments may also reduce production expenses. If toxicity remains an issue even with the low doses required in synergistic antimicrobial combinations, changing amino acids on AMPs has been shown to have strong effects on synergy (74). Moreover, AMPs that can synergize with preexisting AMPs in hosts could be especially potent *in vivo*, due to the activation of natural AMP release by the immune system. In innate immunity, humans express LL-37; therefore, synergies that arise with LL-37, like histones and protegrin 1, would be especially critical to consider for antibiotic applications.

Synergistic antimicrobial combinations are promising candidates that reduce potential bacterial resistance, overcome preexisting resistance to current antibiotics, prevent host toxicity, and increase antimicrobial efficacy. Thus, an improved understanding of mechanisms by which AMPs synergize with other antimicrobials is necessary. Moving forward, the synergistic interactions between AMPs and other antimicrobials will provide promising options to be explored in the development of new antibiotics.

## Author Contributions

LD wrote the initial draft of the manuscript. LD, SG, and AS edited the manuscript. All authors discussed the vision, direction, and scope of the manuscript.

## Conflict of Interest

The authors declare that the research was conducted in the absence of any commercial or financial relationships that could be construed as a potential conflict of interest.
